# Incidence and clinical course of femoral localized periosteal thickening and atypical femoral fracture over a 10-year period in patients with autoimmune inflammatory rheumatic disease

**DOI:** 10.1093/jbmrpl/ziae090

**Published:** 2024-07-10

**Authors:** Hiroe Sato, Naoki Kondo, Yoichi Kurosawa, Eriko Hasegawa, Ayako Wakamatsu, Yukiko Nozawa, Daisuke Kobayashi, Takeshi Nakatsue, Yoko Wada, Junichiro James Kazama, Takeshi Kuroda, Masaaki Nakano, Naoto Endo, Ichiei Narita

**Affiliations:** Division of Clinical Nephrology and Rheumatology, Niigata University Graduate School of Medical and Dental Sciences, 1-757 Asahimachi-Dori, Chuo-ku, Niigata City 951-8510, Niigata, Japan; Health Administration Center, Niigata University, 2-8050 Ikarashi, Nishi-ku, Niigata City 950-2181, Niigata, Japan; Division of Orthopedic Surgery, Niigata University Graduate School of Medical and Dental Sciences, 1-757 Asahimachi-Dori, Chuo-ku, Niigata City 951-8510, Niigata, Japan; Division of Clinical Nephrology and Rheumatology, Niigata University Graduate School of Medical and Dental Sciences, 1-757 Asahimachi-Dori, Chuo-ku, Niigata City 951-8510, Niigata, Japan; Division of Clinical Nephrology and Rheumatology, Niigata University Graduate School of Medical and Dental Sciences, 1-757 Asahimachi-Dori, Chuo-ku, Niigata City 951-8510, Niigata, Japan; Division of Clinical Nephrology and Rheumatology, Niigata University Graduate School of Medical and Dental Sciences, 1-757 Asahimachi-Dori, Chuo-ku, Niigata City 951-8510, Niigata, Japan; Division of Clinical Nephrology and Rheumatology, Niigata University Graduate School of Medical and Dental Sciences, 1-757 Asahimachi-Dori, Chuo-ku, Niigata City 951-8510, Niigata, Japan; Health Administration Center, Niigata University, 2-8050 Ikarashi, Nishi-ku, Niigata City 950-2181, Niigata, Japan; Division of Clinical Nephrology and Rheumatology, Niigata University Graduate School of Medical and Dental Sciences, 1-757 Asahimachi-Dori, Chuo-ku, Niigata City 951-8510, Niigata, Japan; Division of Clinical Nephrology and Rheumatology, Niigata University Graduate School of Medical and Dental Sciences, 1-757 Asahimachi-Dori, Chuo-ku, Niigata City 951-8510, Niigata, Japan; Division of Clinical Nephrology and Rheumatology, Niigata University Graduate School of Medical and Dental Sciences, 1-757 Asahimachi-Dori, Chuo-ku, Niigata City 951-8510, Niigata, Japan; Department of Nephrology and Hypertension, Fukushima Medical University, 1 Hikariga-oka, Fukushima City 960-1295, Fukushima, Japan; Division of Clinical Nephrology and Rheumatology, Niigata University Graduate School of Medical and Dental Sciences, 1-757 Asahimachi-Dori, Chuo-ku, Niigata City 951-8510, Niigata, Japan; Health Administration Center, Niigata University, 2-8050 Ikarashi, Nishi-ku, Niigata City 950-2181, Niigata, Japan; Division of Clinical Nephrology and Rheumatology, Niigata University Graduate School of Medical and Dental Sciences, 1-757 Asahimachi-Dori, Chuo-ku, Niigata City 951-8510, Niigata, Japan; Division of Orthopedic Surgery, Tsubame Rosai Hospital, 633, Sawatari, Tsubame City 959-1228, Niigata, Japan; Division of Clinical Nephrology and Rheumatology, Niigata University Graduate School of Medical and Dental Sciences, 1-757 Asahimachi-Dori, Chuo-ku, Niigata City 951-8510, Niigata, Japan

**Keywords:** atypical femoral fracture, femoral localized periosteal thickening, beaking, glucocorticoid, bisphosphonate

## Abstract

Atypical femoral fracture (AFF) is generally a rare complication of long-term use of bisphosphonate (BP); glucocorticoid (GC) use and Asian race are also risk factors. Femoral localized periosteal thickening (LPT, also termed “beaking”) of the lateral cortex often precedes AFF. This cohort study investigated the incidence of LPT and AFF and their clinical courses over 10 yr in patients with autoimmune inflammatory rheumatic diseases (AIRDs) treated with BP and GC. The study population consisted of 121 patients with AIRDs taking BP and GC. LPT was screened by X-ray, and the LPT shape was evaluated. Prednisolone (PSL) dose was 10 (8–12) mg/d at enrollment and 9 (6–10) mg/d at the last observation. LPT was evident in 10 patients at enrollment and increased linearly to 31 patients (26%) at the last observation. AFF occurred in 9 femurs of 5 patients with LPT. All patients with AFF had bilateral LPT, and the prevalence of pointed type and LPT height were higher in the AFF-positive group than in the AFF-negative group. AFF occurred before BP discontinuation in 2 patients, 1 yr after BP discontinuation in 1, after BP discontinuation followed by 7 yr of alfacalcidol use in 1, and after switching from alfacalcidol to denosumab in 1. The prevalence rates of AFF and LPT associated with long-term BP use with concomitant use of GC (mostly PSL ≥ 6 mg/d) in Japanese patients with AIRD increased over time. The selection of long-term osteoporosis treatment for LPT-positive patients is difficult in some cases.

## Graphical Abstract

**Graphical Abstract:**
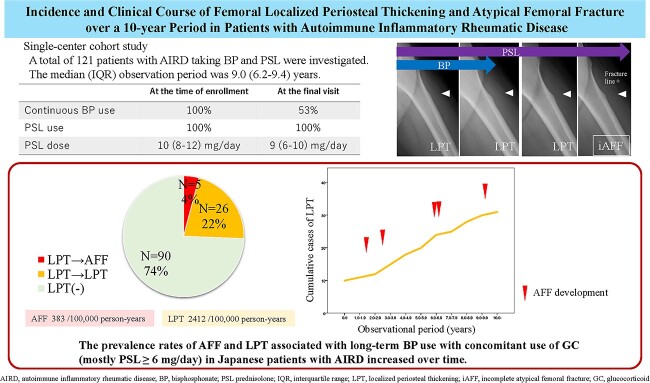

## Introduction

Atypical femoral fractures (AFFs) are stress fractures for which long-term bisphosphonate (BP) use,^1-3^ glucocorticoid (GC) use,^3^^,^^4^ and Asian race^3^^,^^5^ are important risk factors. AFF is generally a rare complication of BP use, which is generally outweighed by the beneficial effects of these drugs in prevention of osteoporotic fractures.^1^ However, greater care is required when using BP for GC-induced osteoporosis (GIOP) in Asian patients due to the overlapping risks of AFF. In a previous study conducted in our hospital, AFF was observed in 5 of 270 (2%) Japanese patients with systemic lupus erythematosus (SLE) requiring long-term prednisolone (PSL) treatment, which was likely to be combined with BP.^6^

Localized periosteal thickening (LPT, also termed “beaking”) of the femoral lateral cortex, which is one of the major features of AFF,^2^ often develops prior to complete AFF (cAFF) or incomplete AFF (iAFF).^7^ We reported an LPT incidence of 8%–10% in a 2-yr follow-up study of patients with autoimmune inflammatory rheumatic disease (AIRD) receiving BP and PSL, and LPT progressed to cAFF in 1 patient.^8^ On the other hand, LPT was observed in 1%–3% of patients with rheumatoid arthritis (RA) regardless of PSL or BP use over a period of 2–3 yr.^9^ Post hoc analysis of these 2 cohorts showed that PSL dose ≥ 5.5 mg/d and long-term BP and PSL use were important factors underlying the difference in LPT frequency.^10^ In general, femoral lateral bowing is a risk factor for AFF, but middle femoral AFF was reported to be associated with bowing, and proximal femoral AFF to be associated with GC.^11^ In our previous studies, most cases of LPT and AFF were seen in the subtrochanteric region, and bowing was not associated with LPT.^8^^,^^9^

The shape of LPT is useful for determining the risk of AFF^12-15^ and, if LPT is high or in cases of pointed type and/or accompanied by prodromal pain, prophylactic fixation is also considered because of the high risk of developing AFF.^15^ Early detection of LPT and discontinuation of BP are thought to be important to prevent AFF, and LPT was shown to improve after BP withdrawal in some cases.^13-15^ Furthermore, BP drug holiday is also considered for prevention of LPT and AFF.^13^^,^^16^^,^^17^ Although there is some consensus regarding drug discontinuation after long-term use of BP in patients with primary osteoporosis, the safety in GIOP has not been adequately studied.

This single-center observational study was performed to clarify the incidence of LPT and AFF, and the change in LPT shape and the sequence of osteoporosis treatment, in patients with AIRD treated with BP and GC over a 10-yr period.

## Materials and methods

### Study protocol

Our previous prospective study with a 2-yr observation period enrolled 125 patients with AIRDs taking BP and PSL between September 2011 and January 2012.^8^ LPT was found in 10% of patients with AIRD using PSL and BP over 2-yr period, and that X-ray images of the bilateral femurs and hip joints were helpful for detecting latent LPT.^8^ Therefore, annual X-ray and determination of BMD using DXA along with monitoring of bone metabolic markers were performed in most patients. This study retrospectively investigated the incidence and clinical courses of LPT and AFF after the previous study until October 2021 and analyzed them over a 10-yr period. Of the 125 AIRD patients who participated in the previous cohort study taking BP and PSL, 4 were examined only at their initial presentation and were excluded from this study. The remaining 121 patients were included in this study; these patients were diagnosed with SLE (*n* = 69, 57%), RA (*n* = 17, 14%), vasculitis (*n* = 14, 12%), mixed connective tissue disease (*n* = 8, 7%), and others (*n* = 13, 11%). Medical history and treatments were reviewed through examination of medical records. Diabetes mellitus was diagnosed based on prescription of any hypoglycemic drug. Osteoporotic fractures and avascular necrosis of the femoral head (ANFH) were determined through examination of medical records and X-rays. The study protocol was approved by the ethics committee of Niigata University (Niigata, Japan; no. 2021-0140), and the study was performed in accordance with the Declaration of Helsinki. Because this was an observational study using existing information, study implementation information was published on the website and written consent was not obtained from individual patients.

### Height and shape of LPT

LPT of the lateral cortex was assessed by X-ray evaluation of the bilateral femurs and hip joints. All X-rays were assessed serially by 2 rheumatologists blinded to the patient data. LPT was defined as localized periosteal thickening of the lateral cortex, which is also termed “beaking.” AFF was diagnosed according to the revised case definition of AFF.^2^ Briefly, AFF is located along the femoral diaphysis and is accompanied by at least 4 of the 5 following: the fracture is associated with minimal or no trauma,; the fracture line originates at the lateral cortex and is substantially transverse in its orientation; complete fractures extend through both cortices and may be associated with a medial spike, while incomplete fractures involve only the lateral cortex; the fracture is noncomminuted or minimally comminuted; and localized periosteal or endosteal thickening of the lateral cortex is present at the fracture site (“beaking” or “flaring”).^2^ In this study, cases with evident dislocation were classified as cAFF. Cases with an obvious fracture line penetrating from the tip of LPT on X-rays without dislocation or when accompanied by severe prodromal pain were classified as iAFF.

The height and shape of LPT were evaluated as described previously (Supplemental Figure S1).^9^^,^^15^ Briefly, LPT height was defined as the distance from the tip of the LPT to the original line of the femoral lateral cortex, and cases where the margin lay inside the lines drawn from the tip of the LPT to the upper and lower points of intersection of the lateral cortex and the margin of the LPT were classified as “pointed type.”

### Bone turnover markers

Serum bone alkaline phosphatase (BAP), type I collagen cross-linked N-terminal telopeptide (sNTx), undercarboxylated osteocalcin (ucOC), and intact PTH levels were measured. Urine deoxypyridinoline levels were also measured. Serum 25(OH)D levels were quantified by radioimmunoassay (Diasorin, Stillwater, MN, USA), and serum pentosidine levels were determined by enzyme-linked immunosorbent assay (ELISA) (Fushimi Pharmaceutical, Kagawa, Japan) at enrollment. The normal ranges of sNTx were 7.5–16.5 nmol BCE/L for premenopausal women, 10.7–24.0 nmol BCE/L for postmenopausal women, and 9.5–17.7 nmol BCE/L for men. Those of BAP were 2.9–14.5 μg/L for premenopausal women, 3.8–22.6 μg/L for postmenopausal women , and 3.7–20.9 μg/L for men.

### Statistical analysis

Most data are expressed as the median (interquartile range) or as numbers with percentages in parentheses. Missing values were analyzed as missing values. The Mann–Whitney U-test was used for continuous variables, whereas Fisher’s test or the χ^2^ test was used for comparison of categorical variables. All statistical analyses were performed using SPSS (ver. 27; SPSS, Armonk, NY, USA). In all analyses, *p* < 0.05 was taken to indicate statistical significance.

## Results

### Characteristics of the study population

The study population consisted of 121 patients (108 women, 89%). At the time of enrollment into the study, the patients were 55 (42–64) yr old, the PSL dose was 10 (8–12) mg/d, the duration of PSL use was 9.1 (5.3–15.3) yr, the duration of BP use was 5.0 (3.0–6.3) yr, and alendronate was used in most cases (92%, *n* = 111). The median observation period was 9.0 (6.2–9.4) yr. At the last observation, the PSL was continued in all cases at a dose of 9 (6–10) mg/d.

### Prevalence of LPT and AFF

**Figure 1:**
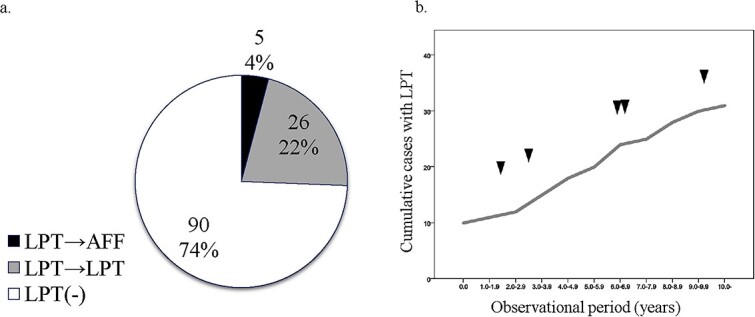
Prevalence of localized periosteal thickening (LPT; also termed “beaking”) of the lateral femoral cortex and atypical femoral fracture (AFF) over the entire observation period (*n* = 121). (a) LPT became AFF in 5 patients (4%). (b) The cumulative number of cases of LPT increased linearly throughout the observation period. Arrowheads indicate development of AFF. In cases of bilateral AFF or LPT, the first to occur was plotted.

**Figure 2:**
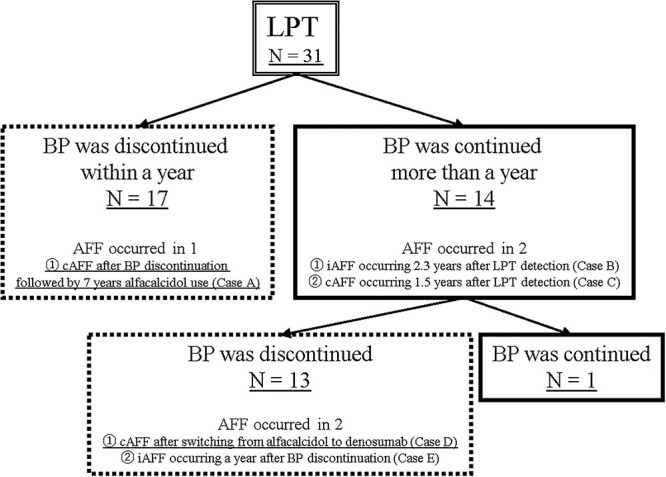
Clinical course of LPT (*n* = 31). Seventeen patients with LPT discontinued bisphosphonate (BP) within 1 yr after LPT detection, and complete atypical femoral fracture (cAFF) occurred after BP discontinuation followed by 7 yr of alfacalcidol use (Case A in Figure 3). Fourteen patients continued BP for >1 yr after LPT detection and AFF occurred in 2; 1 case of incomplete AFF (iAFF) occurring 2.3 yr after LPT detection (Case B in Supplemental Figure S2) and 1 case of cAFF occurring 1.5 yr after LPT detection (Case C in Supplemental Figure S2). Among these 14 patients, only 1 continued BP, with the other 13 discontinuing BP. AFF occurred in the latter 13 with 1 case of cAFF after switching from alfacalcidol to denosumab (Case D in Figure 3) and 1 case of iAFF occurring 1 yr after BP discontinuation (Case E in Supplemental Figure S2).

**Figure 3:**
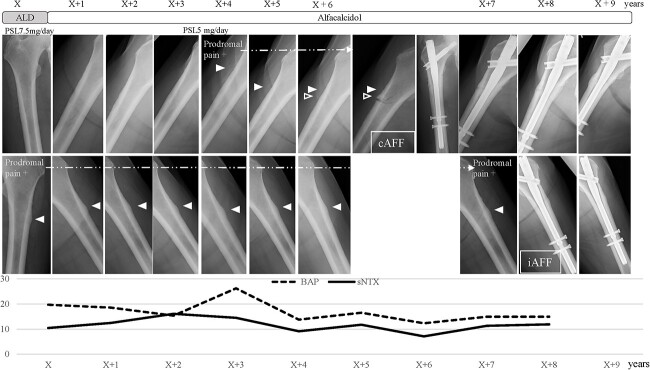
Clinical course of a patient with AFFs (Case A). A 64-yr-old woman with rheumatoid arthritis taking PSL at a dose of 7.5 mg/d was found to have LPT on the left femur at baseline, and had been treated with alendronate for 6.2 yr. The patient had prodromal pain, and alendronate was discontinued within 1 yr after LPT detection. She refused surgical fixation, and TPTD was not used because of accompanying malignant lymphoma. LPT in the right femur appeared in the fifth year, which was also accompanied by prodromal pain. In the seventh year, a new small LPT appeared in the right femur distal to the original LPT. Complete AFF occurred at the second LPT in the right femur, and surgical fixation was performed. Six months later, prodromal pain in the left LPT worsened, and surgical fixation was performed (no fracture line was detected, but a diagnosis of clinically iAFF was made). BAP, bone alkaline phosphatase; cAFF, complete atypical femoral fracture; iAFF, incomplete atypical femoral fracture; sNTX, serum type I collagen cross-linked N-terminal telopeptide. The vertical axis represents BAP (U/L) and sNTX (nmol BCE/L).

**Figure 4:**
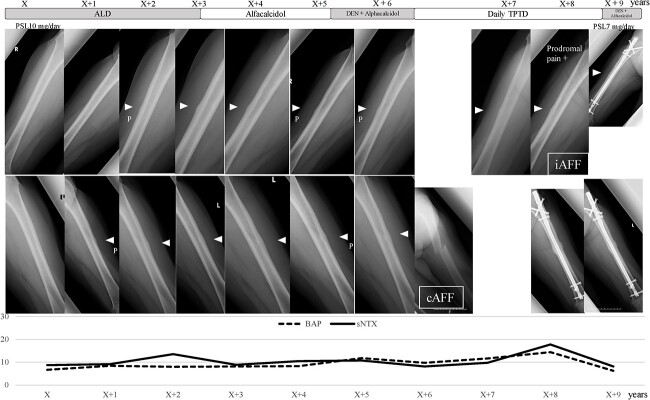
Clinical course of a patient with AFFs (Case D). A 66-yr-old woman with microscopic polyangiitis taking prednisolone (PSL) at a dose of 10 mg/d had taken alendronate for 5.8 yr at enrollment. LPT was detected in the left femur in the second year and LPT was detected in the right femur in the third year. Alendronate was changed to alfacalcidol, but denosumab (DEN) was started because of BMD deterioration (femoral neck T-score − 3.4). One year after starting DEN, a fall resulted in AFF. Surgical fixation was performed and teriparatide (TPTD) was started. Eight months after the surgery, prodromal pain appeared on the right side, and iAFF was treated by surgical fixation. DEN was restarted after the surgery. BAP, bone alkaline phosphatase; cAFF, complete atypical femoral fracture; iAFF, incomplete atypical femoral fracture; P, pointed type; sNTX, serum type I collagen cross-linked N-terminal telopeptide. The vertical axis represents BAP (U/L) and sNTX (nmol BCE/L).

LPT was evident in 10 patients at enrollment and increased linearly by 2 (1–3) patients per yr during the follow-up period. Finally, LPT was observed in 31 patients (26%) and 47 femurs during the entire observation period (Figure 1). All patients with LPT had been treated with BP when LPT was discovered. Of 47 femurs of 31 patients with LPT, AFF occurred in 9 femurs of 5 patients. Three femurs had cAFF and 6 femurs had iAFF, and 4 femurs were treated surgically (Figures 2–4 and Supplemental Figure S2). The prevalence of LPT after commencement of BP was 2412 per 100 000 person-years and that of AFF was 383.

### Comparison of LPT-positive and LPT-negative patients

The characteristics of patients at enrollment were compared between the LPT-positive and LPT-negative groups throughout the entire observation period (Table 1). Age, sex, duration of BP use, PSL dose, and BMD at enrollment were not different between LPT-positive and LPT-negative groups. sNTX was lower in the LPT-positive group than in the LPT-negative group, but the level was within the normal range in most patients. There were no differences in other bone metabolic markers between the 2 groups. The prevalence of osteoporotic fracture and ANFH was not different between LPT-positive and LPT-negative groups. The observation period was longer in the LPT-positive group than in the LPT-negative group, which may have been due to careful monitoring of LPT. The daily PSL dose at the last observation was not different between LPT-positive and LPT-negative groups (10 [6.9–10] vs. 8 [5.8–10] mg, respectively, *p* = 0.401).

**Table 1:** Comparison of patient characteristics at enrollment between LPT-negative and LPT-positive groups throughout the observation period.

	LPT (−)		LPT (+)		*p*
	*n*	Median (IQR) or *n* (%)	*n*	Median (IQR) or *n* (%)	
**Age, yr**	90	59 (43–65)	31	51 (41–60)	0.075
**Women, *n* (%)**	90	78 (87)	31	30 (97)	0.180
**Underlying disease, *n* (%)**	90	SLE, 47 (52); MCTD, 6 (7); RA, 15 (17); vasculitis, 11 (12); myositis, 5 (6); others, 6 (7)	31	SLE, 22 (71); MCTD, 2 (6); RA, 2 (6); vasculitis, 3 (10); myositis, 1(3); others, 1(3)	
**Disease duration, yr**	90	10.2 (5.7–17.9)	31	12.7 (8.9–19.5)	0.191
**Diabetes mellitus, *n* (%)**	90	10 (11)	31	5 (16)	0.530
**PSL dose, mg/d**	90	10.0 (7.5–12.0)	31	10.0 (10.0–12.5)	0.162
**Duration of PSL use, yr**	90	8.5 (4.3–15.5)	31	9.8 (6.9–19.1)	0.204
**Alendronate use, *n* (%)**	90	82 (91)	31	29 (94)	1.000
**Duration of BP use, yr**	90	4.7 (2.9–6.3)	31	5.8 (3.9–6.7)	0.106
**Active form of VD use, *n* (%)**	90	29 (32)	31	6 (20)	0.251
**Calcium preparation use, *n* (%)**	90	32 (23)	31	9 (29)	0.630
**eGFR, mL/min/1.73 m^2^**	90	76.8 (65.7–93.6)	31	74.0 (60.1–85.8)	0.273
**Serum adjusted calcium, mg/dL**	90	9.1 (8.7–9.3)	31	9.1 (8.9–9.3)	0.284
**Bone alkaline phosphatase, U/L**	89	8.6(6.7–10.9)	31	8.4 (6.3–10.5)	0.792
**sNTX, nmol BCE/L**	**89**	**10.3 (9.1–12.1)**	**31**	**9.2 (8.2–10.8)**	**0.019**
**Urine deoxypyridinoline, nmol/mmol·Cr**	87	4.5 (3.4–6.9)	29	4.0 (3.3–4.8)	0.172
**ucOC, ng/mL**	88	0.75 (0.41–1.5)	30	0.56 (0–1.4)	0.496
**Intact PTH, pg/mL**	89	32 (23–43)	31	27 (23–40)	0.187
**25(OH)D, ng/mL**	81	21.8 (16.5–29.4)	25	22.1 (16.5–29.1)	0.947
**Serum pentosidine, μg/mL**	81	0.027 (0.020–0.034)	25	0.023(0.020–0.030)	0.487
**CRP, mg/dL**	89	0.08 (0.02–0.23)	31	0.07(0.02–0.27)	0.928
**Femoral T-score (right)**	84	−1.3 (−2.1, −0.6)	29	−0.9 (−1.7, −0.2)	0.118
**Femoral T-score (left)**	81	−1.5 (−2.0, −0.8)	25	−1.0 (−1.8, −0.5)	0.124
**Lumbar T-score**	88	−0.9 (−2.1, 0.2)	29	−0.6 (−1.6, −0.2)	0.238
**Osteoporotic fracture at enrollment, *n* (%)**	90	5 (6)	31	1 (3)	1.000
**Osteoporotic fracture during observation period, *n* (%)**	90	13 (14)	31	1 (3)	0.113
**ANFH at enrollment, *n* (%)**	90	7 (8)	31	8 (0)	0.189
**ANFH during observation period, *n* (%)**	90	1 (1)	31	2 (7)	0.152
**Observation period, yr**	**90**	**9.0 (5.3–9.3)**	**31**	**9.3 (9.0–9.7)**	**0.003**
**PSL dose at the last observation, mg/d**	90	8 (5.8-10)	31	10 (6.9-10)	0.401

Abbreviations: ANFH, avascular necrosis of the femoral head; BP, bisphosphonate; CRP, C-reactive protein; eGFR, estimated glomerular filtration rate; LPT, femoral localized periosteal thickening; MCTD, mixed connective tissue disease; PSL, prednisolone; RA, rheumatoid arthritis; SLE, systemic lupus erythematosus; sNTX, serum type I collagen cross-linked N-terminal telopeptide; ucOC, undercarboxylated osteocalcin; VD, vitamin D.

### Comparison of AFF-positive and AFF-negative patients in the LPT-positive group

Nine femurs in 5 patients progressed to AFF among 47 femurs in 31 patients with LPT. Table 2 shows a comparison of patient characteristics at the time of LPT detection between AFF-positive and AFF-negative groups. Age, sex, duration of BP use, PSL dose, BMD, and the prevalence of osteoporotic fracture and ANFH were not different between the 2 groups. Although not statistically significant, all patients with AFF had used alendronate and none had used the active form of vitamin D. sNTX levels were significantly lower in the AFF-positive group than in the AFF-negative group. All AFF-positive patients had bilateral LPT, which was significantly higher than the prevalence in AFF-negative patients. The rate of BP discontinuation within 1 yr after the first detection of LPT was not significantly different between the AFF-positive and AFF-negative groups, but 80% of patients (*n* = 4) continued BP after LPT detection in the AFF-positive group. The daily PSL dose at the last observation did not differ between AFF-positive and AFF-negative groups (7.5 [5.0–10] vs. 10 [7.5–10] mg, respectively, *p* = 0.385).

**Table 2:** Comparison of LPT-positive patient characteristics at LPT discovery and LPT shape between AFF-negative and AFF-positive cases

	AFF(-)	AFF(+)	*p*
	26 patients	5 patients	
**Age, yr**	53.5 (47.0–60.5)	65.0 (47.5–68.0)	0.387
**Women, *n* (%)**	25 (96)	5 (100)	1.000
**Underlying disease, *n* (%)**	SLE, 21 (81); RA, 1 (4); vasculitis, 2 (8); myositis, 1 (4); others, 1(4)	SLE, 1 (20); MCTD, 2 (40); RA, 1 (20); vasculitis, 1 (20)	
**Duration of PSL use, yr**	17.3 (9.3–24.1)	7.4(7.0–28.1)	0.417
**PSL dose, mg/d**	10 (8.8–11.1)	10 (8.8–11.3)	0.938
**Duration of BP use, yr**	9.6 (6.3–12.5)	6.9 (5.7–9.4)	0.235
**Alendronate, *n* (%)**	21 (81)	5 (100)	0.560
**Minodronate, *n* (%)**	4 (15)	0 (0)	1.000
**Denosumab, *n* (%)**	1(4)	0 (0)	1.000
**Active form of VD use, *n* (%)**	8 (31)	0 (0)	0.291
**eGFR, mL/min/1.73 m^2^**	62 (60–84)	79 (68–84)	0.331
**Serum calcium, mg/dL**	9.4 (9.2–9.6)	9.6 (9.2–9.8)	0.658
**Bone alkali phosphatase, U/L**	7.7 (6.5–10.9)	10.2 (9.1–16.7)	0.091
**sNTX, nmol BCE/L**	**10.3 (9.5–13.1)**	**8.9 (8.0–9.8)**	**0.022**
**Urine deoxypyridinoline, nmol/mmol·Cr**	4 (3.3–5.2)	3.2 (2.3–3.8)	0.065
**Femoral T-score (right)**	−1.2 (−1.9, −0.3)^a^	−1.5 (−2.4, −0.4)	0.634
**Femoral T-score (left)**	−1.2 (−2.1, −0.6)^b^	−1.3 (−2.1, −0.3)	1.000
**Lumbar T-score**	−0.6 (−1.6, 0.6)^c^	−0.3 (−1.1, 0.7)	0.746
**Osteoporotic fracture at enrollment, *n* (%)**	1 (4)	0 (0)	1.000
**Osteoporotic fracture during observation period, *n* (%)**	1 (4)	0 (0)	1.000
**ANFH at enrollment, *n* (%)**	0 (0)	0 (0)	n.d.
**ANFH during observation period, *n* (%)**	2 (8)	0 (0)	1.000
**Bilateral LPT through observation period, *n* (%)**	**11 (42)**	**5 (100)**	**0.043**
Only in right femur^*^, *n*	16 → 12	1 → 0	
Only in left femur^*^, *n*	4 → 3	2 → 0	
Bilateral femur^*^, *n*	6 → 11	2 → 5	
**BP discontinuation within a year after first LPT detection, *n* (%)**	15 (63)^d^	1 (20)	0.144
**PSL dose at the last observation, mg/d**	10 (7.5-10)	7.5 (5-10)	0.385
	38 femurs	9 femurs	
**Subtrochanteric region, *n* (%)**	28(74)	7(78)	1.000
**Pointed type, *n* (%)**	**2(5)**	**5(56)**	**0.001**
**Prodromal pain, *n* (%)**	**2(5)**	**4(44)**	**0.009**
**Height when discovered, mm**	**1.3(0.8–1.6)**	**2.3(1.3–2.8)**	**0.022**
**Max height, mm**	**1.6(1.3–2.4)^e^**	**3.0(2.2–3.3)^f^**	**0.001**

a, *n* = 24; b, *n* = 19; c, *n* = 25; d, *n* = 24; e, *n* = 35; f, *n* = 8.

Two patients were excluded because 1 yr follow-up was impossible after the first LPT detection(d).

Max height was evaluated excluding femurs evaluated only at discovery (e,f).

^*^Patient number on discovery → Patient number through the entire observation period.

Abbreviations: AFF, atypical femoral fracture; ANFH, avascular necrosis of the femoral head; BP, bisphosphonate; eGFR, estimated glomerular filtration rate; LPT, femoral localized periosteal thickening; MCTD, mixed connective tissue disease; n.d., not determined; PSL, prednisolone; RA, rheumatoid arthritis; SLE, systemic lupus erythematosus; sNTX, serum type I collagen cross-linked N-terminal telopeptide; VD, vitamin D.

The femurs with LPT were then compared between the AFF-positive and AFF-negative groups (Table 2). LPT was mostly subtrochanteric (74%, *n* = 35) and there was no difference between the 2 groups. The prevalence rates of pointed type and prodromal pain were higher in the AFF-positive group than the AFF-negative group, but prodromal pain was detected in only 13% of 47 femurs. Height at the time of LPT detection and maximum height of LPT were both higher in the AFF-positive group than the AFF-negative group. On the other hand, LPT in 2 femurs of 2 patients gradually improved and disappeared after BP discontinuation (Supplemental Figure S3).

### Clinical course of LPT-positive patients

Figure 2 shows the clinical courses of the 31 LPT-positive patients included in the study. BP was discontinued within 1 yr after detection of LPT in 17 patients, and 1 patient progressed to cAFF after discontinuation of BP followed by 7 yr of alfacalcidol use as teriparatide (TPTD) could not be used because of malignant lymphoma (Figure 3, Case A). BP was continued for >1 yr in 14 patients with LPT, and AFF occurred in 2 patients: 1 case of iAFF 2.3 yr after detection of LPT (Supplemental Figure S2, Case B), and 1 case of cAFF 1.5 yr after detection of LPT (Supplemental Figure S2, Case C). Among these 14 patients, AFF occurred in 2 patients after discontinuation of BP: 1 case of cAFF after switching from alfacalcidol to denosumab (DEN) (Figure 4, Case D) and 1 case of iAFF 1 yr after BP discontinuation (Supplemental Figure S2, Case E). BP was continued until the last observation in only 1 LPT-positive patient.

### Osteoporotic fracture and avascular necrosis of the femoral head

Through the entire observation period, BP was continued until the last observation in 53% (*n* = 64) of patients (BP-continuation group), BP was discontinued and changed to the active form of vitamin D or no osteoporotic treatment in 29% (*n* = 35) of patients (BP-discontinuation group), and BP was discontinued and changed to TPTD or DEN, or to the active form of vitamin D followed by BP, DEN, or romosozumab in 18% (*n* = 22) of patients (Supplemental Figure S4). The reasons for BP discontinuation were LPT (*n* = 17), sufficient BMD (*n* = 9), factors associated with osteonecrosis of the jaw (*n* = 6), discontinuation at the patient’s discretion (*n* = 2), and elevation of creatinine kinase (*n* = 1). The duration of BP drug holiday in the BP-discontinuation group was 2.8 (1.5–6.4) yr. At the time of enrollment, the BP-discontinuation group had significantly younger age (51 [38–62] vs. 61 [49–67] yr, respectively, *p* = 0.019), lower sNTX level (9 [8–10] vs. 11 [9–13] nmol BCE/L, respectively, *p* < 0.001), significantly higher lumbar BMD T-score (−0.2 [−1.4, 0.8] vs. −1.1 [−2.0, 0.1], respectively, *p* = 0.018), and significantly higher right femoral neck BMD T-score (−0.8 [−1.3, 0] vs. −1.5 [−2.3, −0.7], respectively, *p* = 0.001) compared to the BP continuation group (Supplemental Table S1).

Osteoporotic fractures occurred in 10 patients (16%) in the BP-continuation group and there were none in the BP-discontinuation group throughout the entire observation period. ANFH occurred in no patients in the BP-continuation group and in 3 (9%) patients in the BP-discontinuation group; only 1 of these 3 cases occurred after BP withdrawal (Supplemental Table S1).

## Discussion

The incidence of LPT and AFF were examined over a period of 10 yr among patients with AIRD using PSL and BP in this study. AFF is generally considered a rare complication of long-term BP use with a reported incidence of 16 cases per 100 000 person-years after using BP for 5 yr and 113 for 10 yr.^1^ In this study, however, the incidence of AFF was very high at 383 cases per 100 000, similar to our previous report of 278 cases among Japanese patients with SLE.^6^ LPT often precedes AFF and potentially exists^7^^,^^8^ and LPT was observed more frequently in AFF associated with BP than in those without BP.^18^ There have been several reports on the prevalence of LPT, with reported rates of 3% in patients with osteoporosis using antiresorptive therapy,^19^^,^^20^ 2% in patients with RA,^9^ and 13%-24% in cancer patients with bone metastasis using high-dose antiresorptive therapy.^21^^,^^22^ In this study, the rate of 8% at enrollment increased linearly to 26% at the last observation by about 2 cases per year, and all patients had used BP and GC when LPT was detected. The main mechanism of AFF associated with BP is thought to be the accumulation of microdamage due to impaired repair by sustained suppression of bone resorption and bone formation.^23-25^ Moreover, accumulated microdamage at the fracture site was reported in a case of subtrochanteric AFFs, although there was no biopsy-proven severely suppressed bone turnover.^26^ In addition to long-term BP use, GC is also a risk factor^3^^,^^4^^,^^27^ that inhibits bone formation, and its chronic use suppresses bone resorption.^28^ Long-term GC use was a risk factor for AFF,^29^ but the GC dose causing for AFF risk has not been analyzed well. For LPT, PSL ≥ 5.5 mg/d was reported to be a risk factor,^10^ and the median dose of PSL in this study was ≥ 5.5 mg/d, i.e. 10 mg at enrollment and 8–10 mg at the last observation, which may explain the high prevalence of LPT. Furthermore, Asian race is also considered a risk factor for AFF.^3^^,^^5^ Therefore, the high prevalence rates of AFF and LPT in this study may have been mainly due to the long-term use of BP and GC (predominantly PSL ≥ 5.5 mg/d) in Japanese patients. On the other hand, femoral lateral bowing is considered a general risk factor for AFF,^30^ but its prevalence is low in patients with AIRD treated with GC and BP and it was not identified as a risk factor in our previous studies.^8^^,^^9^^,^^15^ The weakest point of the femur with respect to tensile stress was determined in both femoral bowing and the lower-extremity axis^31^; future studies need to evaluate lower limb alignment.

Patients with AIRD often receive GC treatment, which is administered together with BP in most cases, based on the GIOP guidelines.^32^^,^^33^ LPT in Japanese AIRD patients was shown to be associated with a higher dose of PSL (≥ 5.5 mg/d), longer use of BP (≥ 5 yr), and longer use of PSL (≥ 7 yr) over an observation period of 2–3 yr, and clinicians should pay particular attention in such cases.^10^ Younger age, diabetes mellitus, and sufficient lumbar T-score were reported to be risk factors for LPT, but sNTX was not examined in this previous study.^10^ In the present study, low sNTX at enrollment was a risk factor for LPT and, at the time of LPT detection, was a risk factor for AFF. As there were no differences in sNTX during the first 2-yr observation period in this cohort,^8^ a longer observation period may make it possible to identify more accurately patients prone to LPT and AFF. Prodromal pain was more common in the AFF-positive group than the AFF-negative group. However, it was seen in only 13% of 47 LPT-positive femurs. In the present study, bilateral LPT was observed in all cases that eventually developed AFF. This observation indicated the need for more careful monitoring with regular X-ray and physical findings in bilateral cases to prevent AFF.

BP should be discontinued in cases where AFF is confirmed, and surgical fixation must be considered in cases with persistent femoral pain.^1^ It may sometimes be difficult to determine whether a patient has iAFF or just LPT, but surgical therapy should be considered in case with iAFF-related findings, such as a radiolucent line at the tip of LPT and prodromal pain.^12^^,^^15^^,^^16^ In LPT, there are cases where the height decreases or disappears with BP withdrawal, so early detection of LPT and early withdrawal of BP may prevent the development of AFF.^13^^,^^14^ Although not statistically significant, only 20% of patients in the AFF-positive group discontinued BP within 1 yr compared with 63% in the AFF-negative group in the present study. AFF risk declined rapidly with BP discontinuation^3^^,^^13^^,^^34^; 1 study showed that BP-related AFF mostly occurred within 1 yr after BP discontinuation,^34^ as in Case E in our study. LPT or AFF occurred in the contralateral femur after BP discontinuation in some cases, and surgical fixation of AFF was recently reported.^35^^,^^36^ Long-term follow-up is warranted after BP discontinuation. Case D had a lower BMD, so DEN was started but cAFF and iAFF finally occurred. It is difficult to strengthen osteoporosis treatment in LPT-positive and AFF-positive cases.^17^ As daily TPTD increases bone turnover, it is expected to work well for treatment of LPT and AFF.^13^^,^^37-41^ The latest GIOP guidelines of the American College of Rheumatology conditionally recommended TPTD over BP for adults with very high fracture risk and adults older than 40 yr with high fracture risk.^32^ However, anti-resorptive treatment for sequential therapy after TPTD is a vexing problem for patients with LPT or AFF. The European Calcified Tissue Society suggested that after TPTD for AFF, BP or DEN be considered if there are bilateral intramedullary pins. A selective estrogen receptor modulator, romosozumab, hormone replacement therapy, or tibolone can be considered, and, if there is evidence of very low bone turnover, BMD and bone turnover markers might be monitored with no follow-up therapy.^39^ The timing of TPTD use is also difficult problem because TPTD can be used for a maximum of 2 yr in a patient’s lifetime.

For postmenopausal osteoporosis, drug holiday after long-term use of BP is recommended in selected cases.^1^ However, as GIOP has a higher-than-usual fracture risk and there is little evidence for the safety of BP withdrawal, the pros and cons of drug withdrawal are not specified in guidelines.^32^^,^^33^ A previous prospective study was performed to examine the efficacy of discontinuing risedronate in patients with SLE, in which 25 patients with SLE using PSL ≥ 2 mg and risedronate ≥ 3 yr discontinued risedronate for 48 wk (age, mean ± SD, 46 ± 11 yr; 92% woman; PSL dose, 8 ± 4 mg/d; duration of risedronate use, 6 ± 2 yr).^42^ In this previous study, lumbar and total hip BMD decreased after risedronate withdrawal, and 1 patient had a clinical lumbar fracture after 20 wk.^42^ Although our study did not prospectively examine withdrawal of BP, the BP-discontinuation group was studied retrospectively in patients with low sNTX and sufficient BMD, and no osteoporotic fractures occurred in this group. Our study was different from the previous study in that most patients were treated with alendronate, which has higher affinity for bone than risedronate.^43^ A population-based, matched, cohort study revealed that hip fracture rates were slightly higher during drug holidays after cessation of risedronate than alendronate.^44^ Further investigations are required, as the safety of drug holidays differs depending on the drug used.

ANFH is another bone-related complication, risk factors for which are GC, alcohol, etc.^45^ BP has been proposed as 1 treatment for ANFH.^45^ However, a meta-analysis of randomized controlled trials indicated no significant efficacy of BP for ANFH.^46^ In the present study, the BP-discontinuation group had a higher frequency of ANFH, but only 1 patient had ANFH after BP withdrawal. Therefore, further research is required.

This study had several limitations. This was an observational study with a small sample size in a single facility. Treatment was selected by the attending physicians. Although medication compliance was not evaluated directly, most patients showed suppressed bone metabolic markers suggesting good compliance rates.

In conclusion, BP is often used for GIOP treatment and prevention, but care is required regarding the occurrence of AFF and LPT, associated with long-term BP use with concomitant use of GC (especially PSL ≥ 5.5 mg/d) in Asian patients, because they showed increased prevalence in this population. As LPT is usually asymptomatic, X-ray screening is an important means of detecting potential LPT in such patients. When LPT is detected, BP should be discontinued as soon as possible, and surgical treatment should be considered in cases accompanied by prodromal pain or in which LPT is of the pointed type or high, or in bilateral cases. The AFF risk declined rapidly with BP discontinuation but is still high within 1 yr after BP withdrawal. Further investigations are necessary to determine the appropriate selection of long-term osteoporosis treatment for LPT-positive patients.

## Supplementary Material

Supplemental_tables_figures_R1_ziae090

## Data Availability

The datasets are available from the corresponding author on reasonable request.
