# Supplementary material for: Incidence and clinical course of femoral localized periosteal thickening and atypical femoral fracture over a 10-year period in patients with autoimmune inflammatory rheumatic disease
Source: JBMR Plus. 2024 Jul 10;8(9):ziae090. doi: 10.1093/jbmrpl/ziae090 (PMC11307327; doi:10.1093/jbmrpl/ziae090)
Supplement: Supplemental_tables_figures_R1_ziae090 [file supplemental_tables_figures_r1_ziae090.pdf]

Incidence and Clinical Course of Femoral Localized Periosteal Thickening and Atypical Femoral Fracture over a 10-year Period in Patients with Autoimmune Inflammatory Rheumatic Disease

Hiroe Sato, Naoki Kondo, Yoichi Kurosawa, Eriko Hasegawa, Ayako Wakamatsu, Yukiko Nozawa, Daisuke Kobayashi, Takeshi Nakatsue, Yoko Wada, Junichiro James Kazama, Takeshi Kuroda, Masaaki Nakano, Naoto Endo, Ichiei Narita

Correspondence to:

Hiroe Sato, M.D., Ph.D.

Health Administration Center, Niigata University

E-mail: hiroes@med.niigata-u.ac.jp

Supplemental Figure 1.

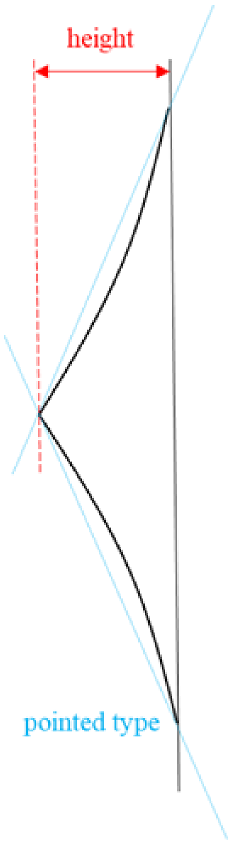

The height of localized periosteal thickening (LPT) was defined as the distance from the tip of the LPT to the original line of the femoral lateral cortex, and “pointed type” was defined as cases where the margin lay inside the lines drawn from the tip of the LPT to the upper and lower points of intersection of the lateral cortex and the margin of the LPT.

Supplemental Figure 2.

Case B.

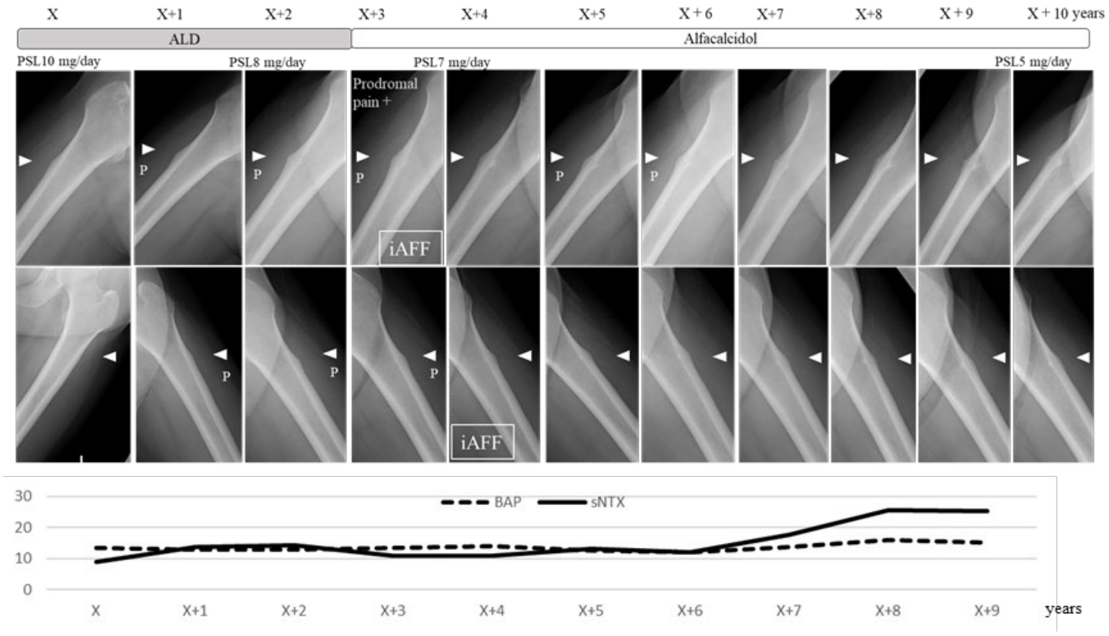

A 51-year-old woman with systemic lupus erythematosus (SLE) taking PSL at a dose of 10 mg/day was found to have latent LPT at baseline and was treated with alendronate for 5.2 years. In the second year, LPT on both sides was of the pointed type. Prodromal pain appeared in the right femur 2.3 years after detection of LPT, and the fracture line was obvious at the tip of the LPT. Alendronate was discontinued. In the fifth year, a fracture line on the left femur was obvious without pain.

The vertical axis represents BAP (U/L) and sNTX (nmolBCE/L).

Case C.

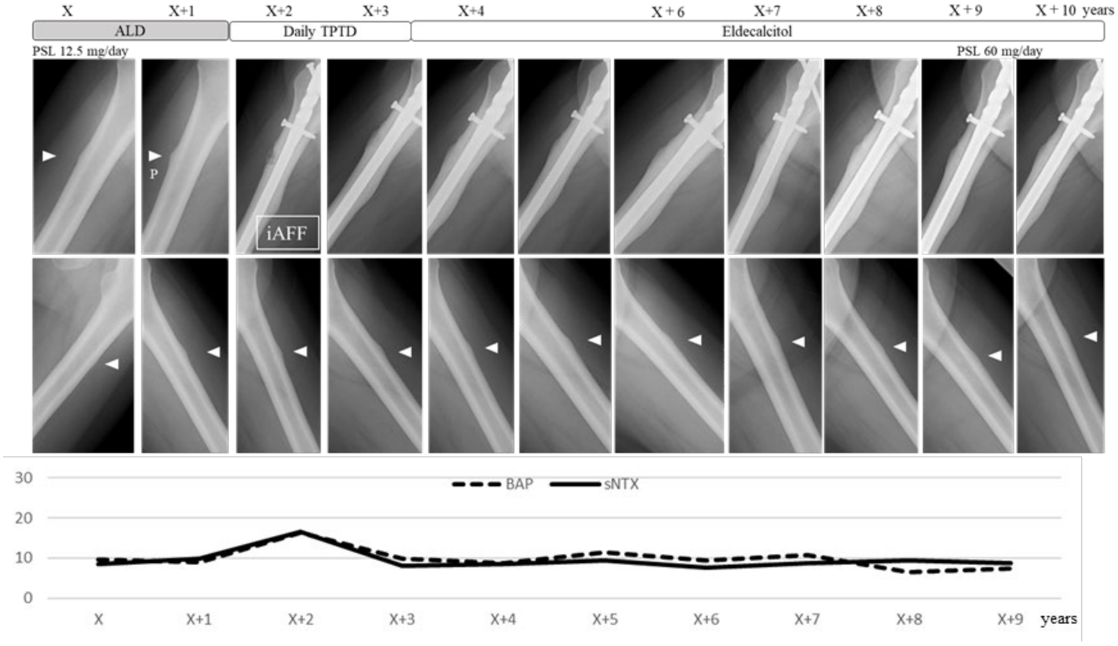

A 42-year-old woman with mixed connective tissue disease (MCTD) taking PSL at a dose of 12.5 mg/day was found to have latent LPT at baseline, and was treated with alendronate for 6.9 years. The LPT of the right femur became pointed type in the second year. She missed the stairs and landed on her right foot, resulting in complete AFF. Surgical fixation was performed and daily TPTD was started.

The vertical axis represents BAP (U/L) and sNTX (nmolBCE/L).

Case E.

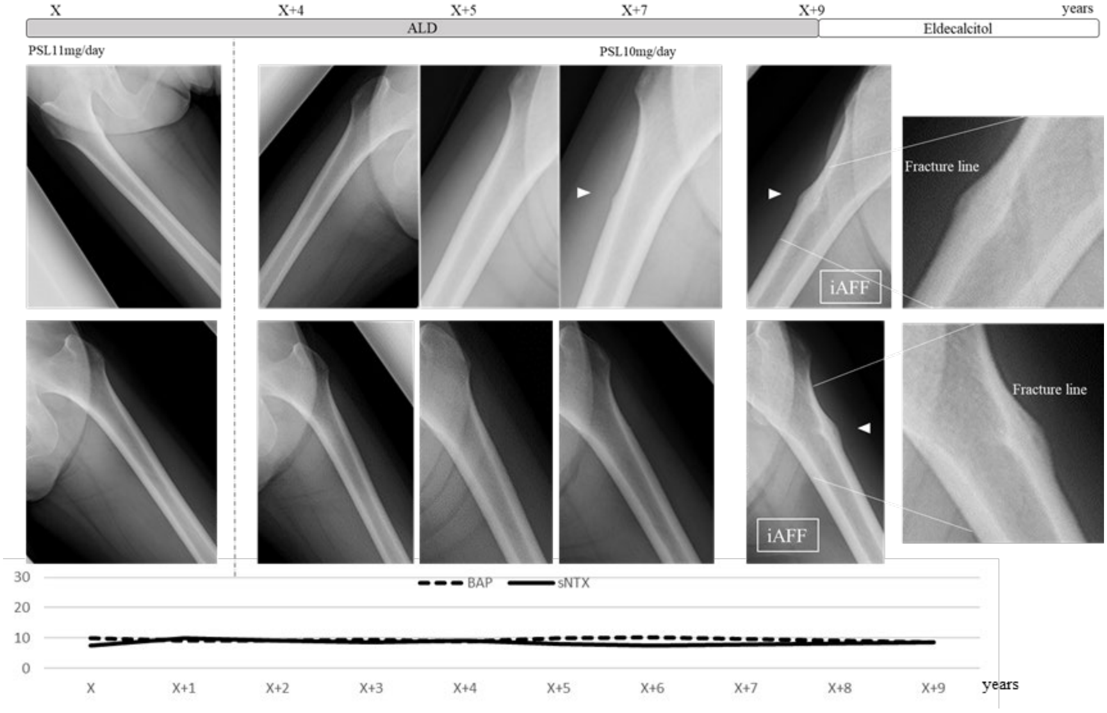

A 62-year-old woman with MCTD. LPT appeared in the right femur, in the eighth year, when she had taken alendronate for 11.8 years and was taking PSL at a dose of 10 mg/day. Alendronate was discontinued but LPT with fracture line appeared in the tenth year.

The vertical axis represents BAP (U/L) and sNTX (nmolBCE/L).

Supplemental Figure 3.

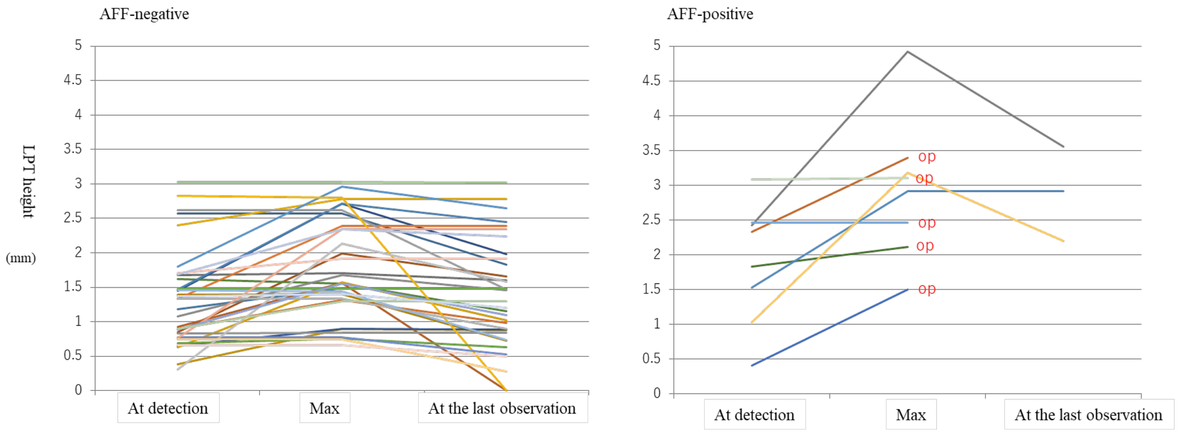

LPT height was plotted at detection, maximum, and the last observation in AFF-negative and AFF-positive groups. Of 47 LPT-positive femurs, 4 were excluded because they could only be evaluated at the time of discovery; n = 35 in the AFF-negative group and n = 8 in AFF-negative group. Two LPT disappeared at the last observation.

AFF, atypical femoral fracture; LPT, localized periosteal thickening; op, surgery.

Supplemental Figure 4.

**The reasons for discontinuation of BP**	
LPT	17
Factors associated with osteonecrosis of the jaw	6
Elevation of creatinine kinase	1
Discontinuation at the patients' own discretion	2
Good BMD	9
	**35**

**After discontinuation of BP**	
DNS	5
TPTD	7
VD→BP	2
VD→DNS	3
VD→TPTD	1
VD→ROM	4
	**22**

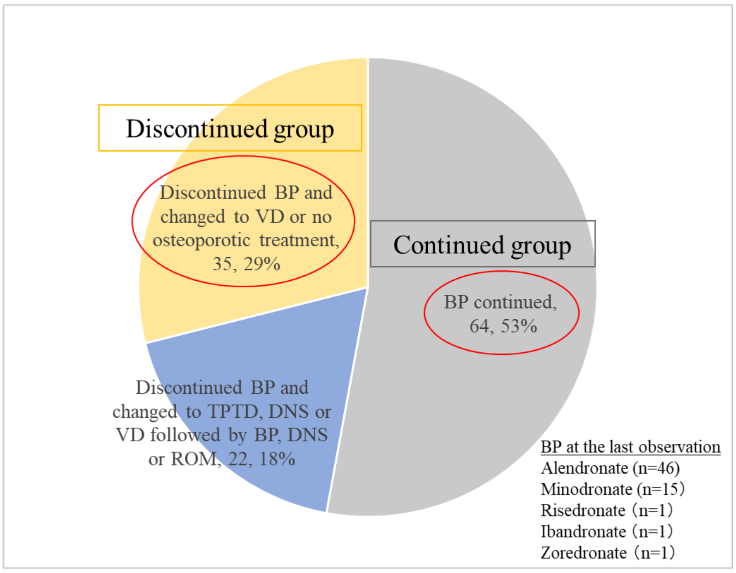

Throughout the entire observation period, BP was continued in 64 patients (53%) before the last observation (BP-continuation group). BP was discontinued in 35 patients (29%) and changed to VD or no osteoporotic treatment (BP-discontinuation group). In the remaining 22 patients (18%), BP was discontinued and changed to TPTD, DEN, or VD followed by BP, DEN, or ROM. The reasons for discontinuation of BP are shown in the orange table.

BMD, bone mineral density; BP, bisphosphonate; DEN, denosumab; LPT, localized periosteal thickening; ROM, romosozumab; TPTD, teriparatide; VD, active form of vitamin D

Supplemental Table 1.

Comparison Between BP-Continuation and BP-Discontinuation Groups

		BP-continuation group (n = 64)	BP-discontinuation group (n = 35)	p
	**LPT-positive**	**4 (6)**	**17 (49)**	**< 0.001**
	**AFF-positive**	**0 (0)**	**3 (9)**	**0.042**
	**Observation period, years**	**9 (4–9)**	**9 (9–10)**	**0.003**
	Women, *n* (%)	55 (86)	32 (91)	0.531
At enrollment	**Age, years**	**61 (49–67)**	**51 (38–62)**	**0.019**
	Duration of PSL use, years	11 (4–16)	8 (5–14)	0.693
	Duration of BP use, years	4 (3–6)	6 (3–7)	0.209
	PSL dose, mg/day	10 (8–12)	10 (9–11)	0.660
	eGFR, mL/min/1.73 m^2	73 (64–87)	81 (67–94)	0.108
	**sNTX, nmolBCE/L**	**11 (9–13)a**	**9 (8–10)**	**< 0.001**
	BAP, U/L	9 (7–11)a	8 (6–10)	0.133
	**Lumbar T-score**	**−1.1 (−2.0, 0.1)a**	**−0.2 (−1.4, 0.8)a**	**0.018**
	**Femoral neck T-score (right)**	**−1.5 (−2.3, −0.7)b**	**−0.8 (−1.3, 0)**	**0.001**
	**Femoral neck T-score (left)**	**−1.6 (−2.2, 0.3)c**	**−1.0 (−1.5, −0.2)d**	**0.001**
	PSL dose at the last observation, mg/day	9 (6–10)	9 (5–10)	0.956
	Osteoporotic fracture, *n* (%)	10 (16)<br>Vertebral fracture (n=8), femoral neck fracture (n=1), pubic bone fracture (n=1)	0 (0)	0.013
	ANFH, *n* (%)	0 (0)	3 (9)<br>ANFH occurred in only one patient after BP discontinuation	0.042

Numbers of missing values: a, *n* = 1; b, *n* = 7; c, *n* = 10; d, *n* = 2.

AFF, atypical femoral fracture; ANFH, avascular necrosis of the femoral head; BAP, bone alkaline phosphatase; BP, bisphosphonate; eGFR, estimated glomerular filtration rate; LPT, localized periosteal thickening; PSL, prednisolone; sNTX, serum type I collagen cross-linked N-terminal telopeptide.
